# Intrarenal pressure detection during flexible ureteroscopy with fiber optic pressure sensor system in porcine model

**DOI:** 10.1038/s41598-024-60080-z

**Published:** 2024-04-24

**Authors:** Junjie Bai, Yangjian Chi, Tong Shangguan, Jun Lin, Yushi Ye, Jianfeng Huang, Yahui Wen, Rong Liu, Ru Chen, Weizhong Cai, Jianhui Chen

**Affiliations:** 1 Department of Urology, Fujian Medical University Union Hospital, Fuzhou, China; 2 Department of Urology, Zhenghe County Hospital, Nanping, China; 3 Department of Breast Surgery, Fujian Medical University Union Hospital, Fuzhou, China

**Keywords:** Intrarenal pressure, Fiber-optic, Ureteroscopy, Surgical position, Renal calyx, Outcomes research, Translational research, Diseases, Medical research, Risk factors, Urology

## Abstract

To validate the feasibility of a fiber-optic pressure sensor-based pressure measurement device for monitoring intrarenal pressure and to analyze the effects of ureteral acess sheath (UAS) type, surgical location, perfusion flow rate, and measurement location on intrarenal pressure (IRP). The measurement deviations and response times to transient pressure changes were compared between a fiber-optic pressure sensing device and a urodynamic device IRP in an in vitro porcine kidney and in a water tank. Finally, pressure measurements were performed in anesthetized female pigs using fiber-optic pressure sensing device with different UAS, different perfusion flow rates, and different surgical positions at different renal calyces and ureteropelvic junctions (UPJ). According to our operation, the result is fiber optic pressure sensing devices are highly accurate and sensitive. Under the same conditions, IRP varied among different renal calyces and UPJ (*P* < 0.05). IRP was lowest at 50 ml/min and highest at 150 ml/min (*P* < 0.05). Surgical position had a significant effect on IRP (*P* < 0.05). 12/14 Fr UAS had a lower IRP than 11/13 Fr UAS. Therefore fiber optic pressure sensing devices are more advantageous for IRP measurements. In ureteroscopy, the type of ureteral sheath, the surgical position, the perfusion flow rate, and the location of the measurement all affect the intrarenal pressure value.

## Introduction

During the procedure of ureteroscopy, the act of irrigating the renal system serves the purpose of achieving a clear visual field in order to effectively carry out the processes of stone fragmentation and extraction. Nevertheless, it is important to acknowledge that this enhanced visual clarity may be accompanied by an elevation in intrarenal pressure (IRP), potentially resulting in the occurrence of pyelovenous or pyelosinus backflow^1,2^. When associated with urinary tract infections, retrograde flow of fluid from the collecting system into blood vessels or adjacent tissues may cause increased fluid reabsorption, predisposing to infectious complications such as fever and sepsis^3,4^. Prior research has investigated the utilization of ureteral access sheaths (UAS) as a means to mitigate IRP. The accomplishment of this involves facilitating the efflux of flushing fluid to prevent the buildup of pressure in the renal calyx and pelvis. Pressure sensors, such as cardiac pressure wires and bladder pressure sensors, were utilized in these studies. They were inserted into the kidney through ureteral catheters or nephrostomy tubes^5–7^. Hence, it is important to acknowledge that measurements obtained during flexible ureteroscopy (fURS) may not accurately represent the precise conditions, necessitating the use of specialized equipment for replication. Additionally, it is imperative to investigate alternative intraoperative factors that may impact IRP.

Real-time pressure measurements of tendons have been done since the 1980s with fiber optic sensors^8,9^. Over the past decade, they have been used in many different medical applications, ranging from skin contact pressure pads^10^ to cardiovascular systems and even invasive sensors used in urodynamic analysis^11^. A number of researchers have examined the use of fiber optic sensors as a means of monitoring and detecting vital signs. There have been no studies applying fiber-optic sensors to the detection of intrarenal pelvic pressure in ureteroscopic surgery.

Therefore, a more accurate, sensitive and non-invasive method of pressure measurement is needed for ureteroscopy and manipulation. Our team has developed a pressure measurement system based on a fiber optic pressure sensor, and this study will verify the accuracy and sensitivity of this system in animal model experiments for further analysis.

## Results

**Figure 1:**
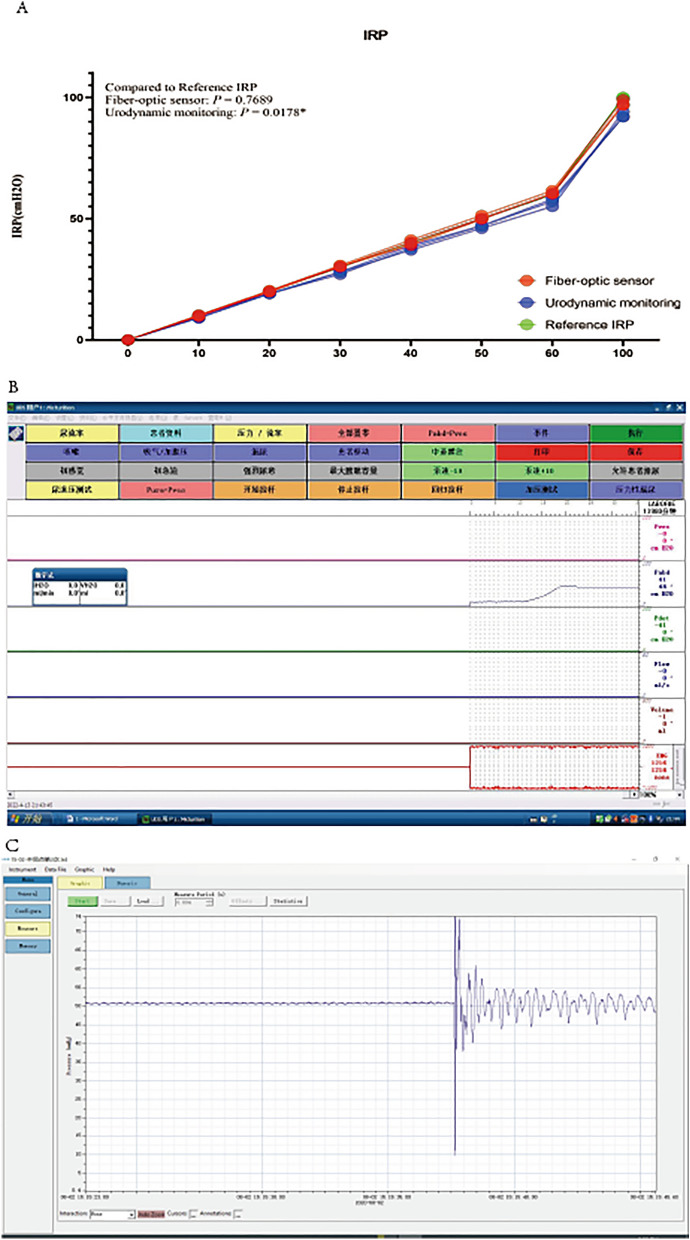
Pressure and response time detection of fiber optic pressure sensor and urodynamic pressure sensor [(**A**): Measured values of fiber-optic pressure sensors and urodynamic pressure sensors versus true values. (**B**): Response time of the urodynamic pressure sensor to a sudden change in pressure. (**C**): Response time of the fiber optic pressure sensor to a sudden change in pressure].

The results of pressure testing at different water depths and the intrarenal pressure (IRP) and response time of ex vivo pig kidneys are summarized in Table 1 and Fig. 1, respectively. When measuring pressure at different water depths, there was a deviation between the pressure values measured by the fiber optic sensor and those measured by the urodynamic system compared to the true values (− 0.10 ± 1.09 vs. − 1.25 ± 2.03, *P* = 0.011), the IRP measured by the fiber optic pressure sensor were closer to the true values. Regarding response time, although there was no statistical difference, the fiber optic pressure sensor demonstrated a shorter response time compared to the urodynamic system (0.3 vs. 0.95, *P* = 0.867).

**Table 1:** Intrarenal pressure analysis in isolated porcine kidneys with fiber-optic pressure sensors and urodynamic instrumentation.

	Fiber optics sensor	Urodynamic monitoring	*P* value
IRP (cm H_2_O)			0.0067*
10 cm	10.088	9.33	
20 cm	20.313	19.00	
30 cm	30.817	27.89	
40 cm	40.700	38.00	
50 cm	51.099	46.56	
60 cm	61.586	56.56	
100 cm	99.025	92.89	
Response time (s)	0.3	0.95	0.867
Bias (s)	− 0.10 ± 1.09	− 1.25 ± 2.03	0.011*

*P < 0.05.

**Figure 2:**
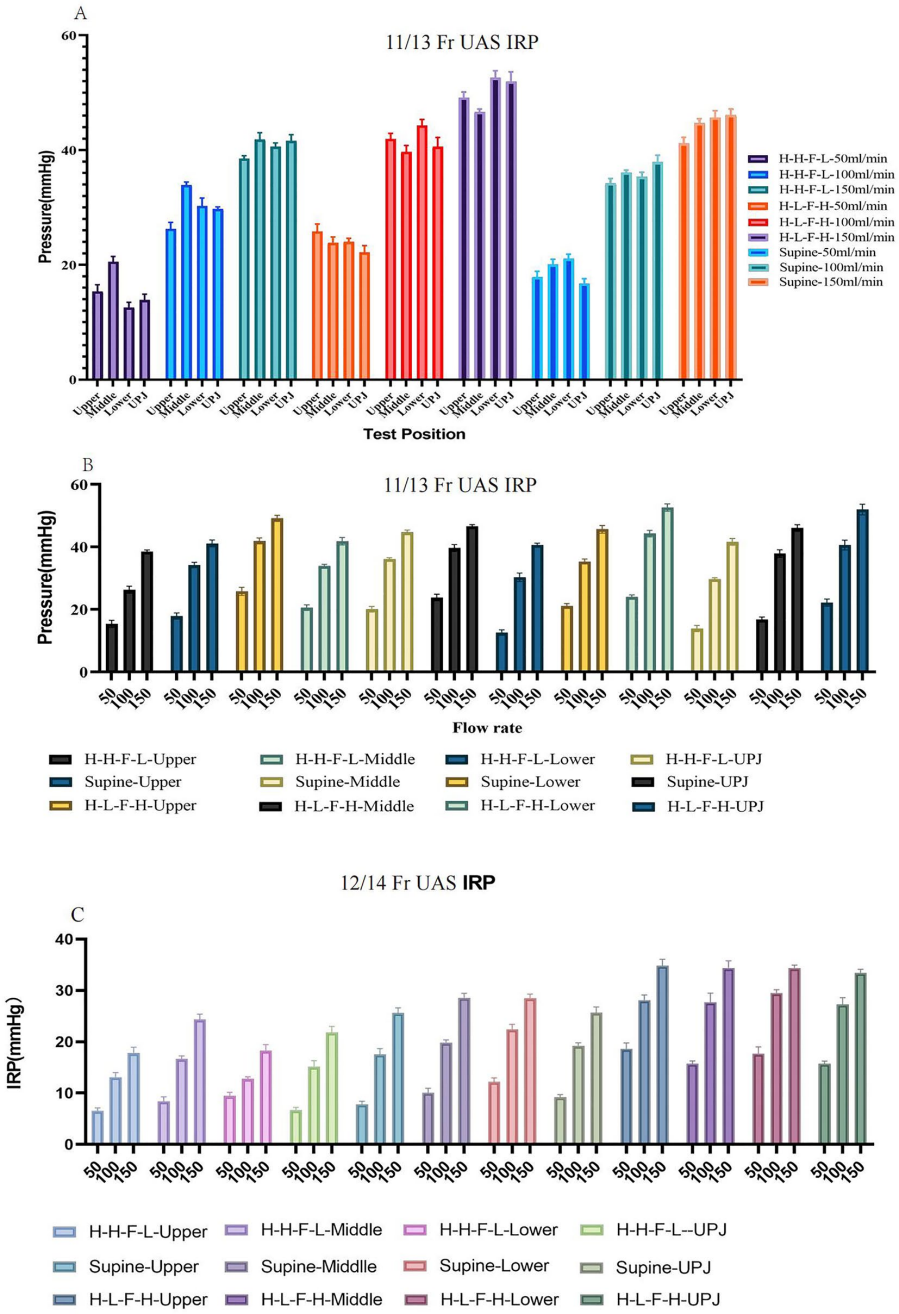
IRP in different types of UAS, surgical positions, irrigation flow rates, and intrarenal pressure measurement locations. [(**A**): IRP in different renal calyces with same surgical positions and same water flow velocities in 11/13 Fr UAS. (**B**): IRP in different water flow velocities with same surgical positions and different renal calyces 11/13 Fr UAS. (**C**): IRP in different water flow velocities with same surgical positions and different renal calyces 12/14 Fr UAS].

**Figure 3:**
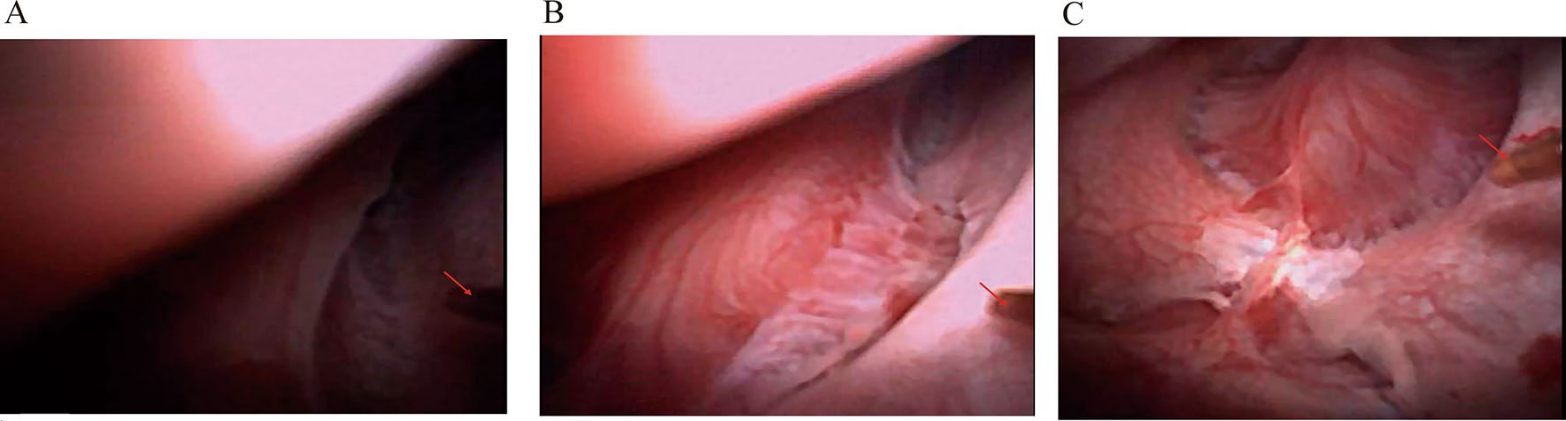
Visual fields of the same surgical position, the same intrarenal manometry position, and different water flow perfusion rates. [(**A**): 50 ml/min. (**B**): 100 ml/min. (**C**): 150 ml/min].

The results obtained using different types of UAS, various surgical positions, irrigation flow rates, and different intrarenal pressure measurement locations are summarized in Tables 2, 3, 4 and 5 and Figs. 2 and 3.

**Table 2:** IRP in different renal calyces with different surgical positions and different water flow velocities in 11/13 Fr UAS.

IRP	H–H–F–L				Supine				H–L–F–H			
	50	100	150	*P*	50	100	150	*P*	50	100	150	*P*
Upper	15.38 ± 1.16	26.29 ± 1.12^a^	38.51 ± 0.51^ab^	< 0.001	17.88 ± 1.00	34.20 ± 0.85^a^	41.13 ± 1.10^ab^	< 0.001	25.82 ± 1.28	41.94 ± 0.96^a^	49.15 ± 0.97^ab^	< 0.001
Middle	20.56 ± 0.90^A^	33.91 ± 0.51^Aa^	41.83 ± 1.19^Aab^	< 0.001	20.08 ± 0.88^A^	36.07 ± 0.45^Aa^	44.67 ± 0.77^Aab^	< 0.001	23.83 ± 1.03^A^	39.70 ± 1.10^Aa^	46.64 ± 0.50^Aab^	< 0.001
Lower	12.59 ± 0.89^AB^	30.27 ± 1.36^ABa^	40.58 ± 0.65^Aab^	< 0.001	21.09 ± 0.77^A^	35.33 ± 0.80^a^	45.66 ± 1.22^Aab^	< 0.001	24.02 ± 0.61^A^	44.28 ± 1.05^ABa^	52.62 ± 1.18^ABab^	< 0.001
UPJ	13.88 ± 1.02^B^	29.73 ± 0.37^ABa^	41.59 ± 1.08^Aab^	< 0.001	16.75 ± 0.82^BC^	37.89 ± 1.20^ABCa^	46.09 ± 1.05^Aab^	< 0.001	22.19 ± 1.16^ABC^	40.60 ± 1.59^Ca^	51.96 ± 1.70^ABab^	< 0.001
*P*	< 0.001	< 0.001	< 0.001		< 0.001	< 0.001	< 0.001	–	< 0.001	< 0.001	< 0.001	–

A: *P* value < 0.05 compared with Upper; B: *P* value < 0.05 compared with Middle; C: *P* value < 0.05 compared with Lower; a: *P* value < 0.05 compared with 50 ml/min; b: *P* value < 0.05 compared with 100 ml/min.

**Table 3:** Intrarenal pressure in different renal calyces with different surgical positions and different water flow velocities 11/13 Fr UAS.

	50				100				150			
	H–H–F–L	Supine	H–L–F–H	*P*	H–H–F–L	Supine	H–L–F–H	*P*	H–H–F–L	Supine	H–L–F–H	*P*
Upper	15.37 ± 1.16	17.88 ± 1.00^a^	25.82 ± 1.28^ab^	< 0.001	26.29 ± 1.12	34.20 ± 0.85^a^	41.94 ± 0.96^ab^	< 0.001	38.51 ± 0.51	41.13 ± 1.10^a^	49.15 ± 0.97^ab^	< 0.001
Middle	20.56 ± 0.91	20.08 ± 0.89	23.83 ± 1.03^ab^	< 0.001	33.91 ± 0.50	36.07 ± 0.45^a^	39.69 ± 1.10^ab^	< 0.001	41.83 ± 1.19	44.67 ± 0.77^a^	46.64 ± 0.50^ab^	< 0.001
Lower	12.59 ± 0.89	21.09 ± 0.77^a^	24.02 ± 0.61^ab^	< 0.001	30.27 ± 1.36	35.33 ± 0.80^a^	44.28 ± 1.04^ab^	< 0.001	40.58 ± 0.65	45.66 ± 1.22^a^	52.61 ± 1.18^ab^	< 0.001
UPJ	13.88 ± 1.01	16.75 ± 0.82^a^	22.19 ± 1.16^ab^	< 0.001	29.73 ± 0.37	37.89 ± 1.20^a^	40.60 ± 1.59^ab^	< 0.001	41.59 ± 1.08	46.09 ± 1.05^a^	51.96 ± 1.69^ab^	< 0.001
*P*	< 0.001	< 0.001	< 0.001		< 0.001	< 0.001	< 0.001		< 0.001	< 0.001	< 0.001	

a: *P* value < 0.05 compared with H–H–F–L; b: *P* value < 0.05 compared with Supine.

**Table 4:** Intrarenal pressure in different renal calyces with different surgical positions and different water flow velocites in 12/14Fr UAS.

	H–H–F–L				Spine				H–L–F–H			
	50	100	150	*P*	50	100	150	*P*	50	100	150	*P*
Upper	6.54 ± 0.58	13.12 ± 0.88^a^	17.84 ± 1.09^ab^	< 0.001	7.83 ± 0.60	17.53 ± 1.15^a^	25.63 ± 0.98^ab^	< 0.001	18.66 ± 1.12	28.11 ± 1.00^a^	34.81 ± 1.29^ab^	< 0.001
Middle	8.43 ± 0.85^A^	16.69 ± 0.54^Aa^	24.35 ± 1.02^Aab^	< 0.001	10.08 ± 0.88^A^	19.86 ± 0.52^Aa^	28.55 ± 0.90^Aab^	< 0.001	15.75 ± 0.52^A^	27.71 ± 1.76^a^	34.38 ± 1.39^ab^	< 0.001
Lower	9.46 ± 0.69^A^	12.78 ± 0.40^Ba^	18.31 ± 1.13^Bab^	< 0.001	12.18 ± 0.80^AB^	22.46 ± 0.95^ABa^	28.50 ± 0.77^Aab^	< 0.001	17.73 ± 1.29^B^	29.53 ± 0.62^Ba^	34.38 ± 0.58^ab^	< 0.001
UPJ	6.69 ± 0.55^BC^	21.80 ± 1.17^ABCa^	15.17 ± 1.17^ABCab^	< 0.001	9.23 ± 0.49^C^	19.25 ± 0.56^ACa^	25.67 ± 1.12^BCab^	< 0.001	15.74 ± 0.51^AC^	27.28 ± 1.32^Ca^	33.41 ± 0.72^ab^	< 0.001
*P*	< 0.001	< 0.001	< 0.001		< 0.001	< 0.001	< 0.001		< 0.001	< 0.001	0.071	

A: *P* value < 0.05 compared with Upper; B: *P* value < 0.05 compared with Middle; C: *P* value < 0.05 compared with Lower; a: *P* value < 0.05 compared with 50 ml/min; b: *P* value < 0.05 compared with 100 ml/min.

**Table 5:** Intrarenal pressure in different renal calyces with different surgical positions and different water flow velocities 12/14 Fr UAS.

	50				100				150			
	H–H–F–L	Spine	H–L–F–H	*P*	H–H–F-L	Spine	H–L–F–H	*P*	H–H–F-L	Spine	H–L–F–H	*P*
Upper	6.54 ± 0.58	7.83 ± 0.60	18.66 ± 1.12^ab^	< 0.001	13.12 ± 0.88	17.53 ± 1.15^a^	28.11 ± 1.00^ab^	< 0.001	17.84 ± 1.09	25.63 ± 0.98^a^	34.81 ± 1.29^ab^	< 0.001
Middle	8.43 ± 0.85	10.08 ± 0.88^a^	15.75 ± 0.52^ab^	< 0.001	16.69 ± 0.54	19.86 ± 0.52^a^	27.71 ± 1.76^ab^	< 0.001	24.35 ± 1.02	28.55 ± 0.90^a^	34.38 ± 1.39^ab^	< 0.001
Lower	9.46 ± 0.69	12.18 ± 0.80^a^	17.73 ± 1.29^ab^	< 0.001	12.78 ± 0.40	22.46 ± 0.95^a^	29.53 ± 0.62^ab^	< 0.001	18.31 ± 1.13	28.50 ± 0.77^a^	34.38 ± 0.58^ab^	< 0.001
UPJ	6.69 ± 0.55	9.23 ± 0.49^a^	15.74 ± 0.51^ab^	< 0.001	21.80 ± 1.17	19.25 ± 0.56^a^	27.28 ± 1.32^ab^	< 0.001	15.17 ± 1.17	25.67 ± 1.12^a^	33.41 ± 0.72^ab^	< 0.001
*P*	< 0.001	< 0.001	< 0.001		< 0.001	< 0.001	< 0.001		< 0.001	< 0.001	0.071	

a: *P* value < 0.05 compared with H–H–F–L; b: *P* value < 0.05 compared with Supine.

All six pigs were comprehensively examined, and it was determined that there was no notable disparity in calyx pressure among the porcine models when subjected to identical test parameters. However, a significant distinction (*P* < 0.05) in pressures was observed between the upper, middle, and lower calyces, as well as the UPJ, under the same test conditions. Notably, no specific pressure location consistently exhibited higher or lower intrarenal pressure compared to the other locations (*P* < 0.05). Consequently, the pressures of the three renal calyces and the UPJ were individually considered for further analysis.

With the 11/13 Fr UAS, the ratio of the outer diameter of the ureteral access sheath to the outer diameter of the ureteroscope is 0.86. IRP increased with increasing water flow rate in the same surgical position and at the same intrarenal manometry position. At the same surgical position and the same water flow perfusion rate, the IRP was different at different intrarenal manometry positions, and there is no pressure measurement position where the IRP value is always at highest value. (*P* < 0.05).In analyzing the effect of surgical position on intrarenal pressure, we found that intrarenal pressure was lowest in the head-high-foot-low position and highest in the head-low-foot-high position (*P* < 0.05). At an irrigation flow rate of 50 ml/min, the intrarenal pressure (IRP) remains within safe limits, but the surgical field is narrow, and there is inadequate lighting. Increasing the flow rate to 100 ml/min improves the surgical field and lighting, but the IRP value almost exceeds safe limits. At a flow rate of 150 ml/min, the surgical field is broad, and lighting is sufficient, but the pressure values exceed safe limits.

With the 12/14 Fr UAS, the ratio of the outer diameter of the ureteral access sheath to the outer diameter of the ureteroscope is 0.79. Measurements were similar to the 11/13 Fr UAS, but the IRP values were below the safety threshold for all conditions except for H–L–F–H and 150 ml/min, where the IRP values exceeded the safety threshold.

## Discussion

The use of fiber optic sensors has been widespread in many applications, including the measurement of chemical parameters, liquid flow and levels, and the detection of gases. Although they possess great advantages such as resistance to electromagnetic interference and small fibers, they have yet to be widely accepted in the medical field^12^. To the best of our knowledge, fiber-optic sensors have never been used for the detection of IRP during ureteroscopic procedures, to the best of our knowledge. Therefore, we propose a pressure measurement system based on fiber-optic sensors herein, which comes with an external pressure sensor for noninvasive estimation of IRP, and it has been demonstrated that this system is feasible from preliminary modeling experiments and porcine kidney experiments. Researchers have tried measuring IRP with miniature pressure sensor wires and UAS integrated with sensors, but no widespread approach has been developed for monitoring IRP in the clinic^13,14^. In the past, only three studies have attempted to measure IRP during real-time RIRS, and only two studies have attempted to control IRP^15–19^. To measure the IRP noninvasively, Shu et al. proposed an irrigation system using syringes and an external pressure sensor^20^. An integrated platform including a pressure monitor, a peristaltic pump, a DC motor negative pressure pump, and computer software was developed by Zhu et al.^21^. There are some limitations with these methods, such as the soft and expensive sensor wires or the inability to reflect intrarenal pressure values in a timely manner in the pressure value collected from the anterior end of the sheath^13^, even cannot be inserted into the operated calyx for manometry. In comparison, it is possible that values from invasive pressure measurements may vary widely in IRPs measured at different locations due to changes in inflow/outflow fluid dynamics caused by percutaneous placement of pressure sensing wires rather than retrograde placement of sensors^22^. In contrast to existing intrarenal pressure testing systems, our manometry system allows for noninvasive assessment of IRP levels, with IRP being digitized, displayed timely, and without worrying about pressure loss variations due to the position of operating instruments in the surgical channel^20,23^. Insertion of the instrument does not increase measurement error because the fiber optic tip is always located at the end of the ureteral lens, not in the channel. In the era of dual-channel ureteroscopy, it is now possible to estimate IRP with high precision when one working channel is used for irrigation and the other for instrumentation. We do not need to worry about pressure changes caused by inserting surgical instruments into the working channel of the flexible ureteroscope. In our study, by comparing with the urodynamic detector, we found that the pressure measurement system based on fiber optic pressure sensor is more accurate, closer to the real value, and more sensitive and responsive to the pressure change, which verifies the feasibility of the system. In general, although surgical instruments can have an impact on perfusion flow, our system still provides urologists with an approximation of IRP in real time to support their clinical decisions.

Whether endoscopic or intrarenal surgery is performed, the importance of pressure cannot be overstated. It is generally accepted that a pressure of approximately 30 mmHg is the ideal threshold below which IRP should be maintained when performing endoluminal surgery, as mentioned in the 2020 European Association of Urology (EAU) guidelines^24^. When the IRP exceeds 30 mmHg, pyelo-venous backflow occurs. This backflow can be life-threatening if infected environments exist. As far as we are aware, this is the first study to examine the feasibility of measuring IRP during f-URS with a fiber. We evaluated IRP in f-URS obtained in different pyelocaliceal locations with 11/13 Fr access sheaths and different operating positions with an anesthetized porcine model. We zeroed the measurement system to atmospheric pressure and then measured the operative pressures. Intraoperatively, we measured and reported the absolute intra-renal pressures, and analyzed the effects of different water flow velocity, different renal calyces, and different surgical positions on IRP. In our preliminary study, it was possible to keep the IRP below 30 mmHg when the flow rate reached 50 mL/min, but it was difficult to maintain high visualization. When the perfusion rate was increased to improve visualization, the IRP exceeded the desired threshold. In cases of combined urinary tract infections, higher flow rates are needed to maintain clear visualization, but also result in increased IRP. It's worth mentioning that this is the first study to examine the effect of surgical position on intrarenal pressure. During flexible ureteroscopy, the surgical position may change the location of the stone, which may affect the treatment outcome^25^. However, there is a lack of literature on whether it changes the IRP. We found that the IRP was lowest in the head-high-foot-low position and highest in the head-low-foot-high position, and that in most cases the pressure was higher in the lower pelvis than in the upper pelvis.

Several studies assessing IRP during URS positioned the pressure transducer within the renal pelvis, while a limited number of studies measured the IRP from a renal calyx^13,21,26^. There were no significant differences in pressure between upper, middle, and lower renal calyces, according to Wang’s study^27^. In our experiment, there was a significant pressure difference between upper, middle, lower renal calyces and UPJ under the same conditions. It may be caused by the reduction in blood flow to the kidneys after death, which reduces kidney tissue elasticity^28,29^. The maximum pressure values were not always in the same renal calyx, and this may be related to the surgical position. Surgeons routinely perform RIRS in the supine position^20^, despite this, it is unclear whether IRP varies with patient positioning^30^. We found that IRP changed with the surgical position. At H–H–F–L, the pressure in the middle renal calyx was maximum. In the supine position, the pressure of the middle and lower renal calyces was maximum, but there was no significant difference between the two. At H–L–F–H, the lower renal calyces had the maximum pressure. This may be because water is retained in the kidneys in different calyces or due to differences in the structure of the renal pelvic system in humans and experimental pigs. However, in contrast, the intrarenal calyceal pressure in H–L–F–H is more likely to exceed the safe one, and the intrarenal calyceal pressure in H–H–F–F is within the safe value. It is true that the change in body position will have an effect on the stone surgery, which the urologist will decide based on the specific circumstances.

Poor drainage would result in a temporary elevation of RPP greater than 30 mm Hg, and frequent high IRO occurrences would cause enough backflow to cause bacteremia and postoperative fever^3^. The diameter of the URS and the diameter of the flexible ureteroscope are also important factors, UASs with smaller sizes are preferable to minimize the chance of ureteral trauma. The smaller sheath will result in a smaller egress gap between the fURS and UAS, causing impaired reflux of flushing fluid and elevated IRP. The use of a ureteral access sheath of 11/13 Fr caliber or larger appears to limit the mean IRP below this threshold in most situations, including the use of pressurized irrigation systems; however, the IRP may be higher than 30 mmHg with the use of additional manual pump/syringe or gravity irrigation > 1.1 m in height^31,32^. In our study, the volume of 50 ml/min of irrigation water when using a sheath of 11/13 Fr resulted in an IRP within the threshold, but with a limited surgical field of view. However, when the water flow was increased, the surgical field was clear and the IRP easily exceeded the threshold.

The ratio of the diameter of the flexible ureteroscope to the diameter of UAS is decisive. It has been shown that a sufficiently large instrument/lock ratio is necessary to avoid elevated renal pressures, which would result in a correspondingly high reflux rate. Therefore, it is recommended to maintain a ratio of 0.75 between the access shaft and the instrument^26^. The advantages of larger sheath diameters are higher irrigation flows, lower intrarenal pressure and supposedly better visibility as well as the possibility of recovering larger fragments, it’s easily to damage the ureter. In our study, the larger ureteral access sheaths resulted in less intrarenal pressure, but difficulties in placing the ureteral access sheaths as well as ureteral injuries occurred during the maneuver.

Paucharda et al. proposed a novel UAS integrating irrigation, aspiration, and IRP measurements, but this requires a larger diameter and whenever IRP measurements need to be taken and debris aspirated, the UAS should be placed above the UPJ, which is inconsistent with its traditional placement, thus increasing the risk of potential damage to the UPJ and ureteroscope^33^. And we are trying to develop a new type of ureteroscope that combines a ureteroscope and a pressure measurement system.First, it can be used without UAS. Secondly, it has the advantage of measuring IRP while treating ureteral stones, which correlates well with the real IRP. Third, it does not take up valuable space within the working channel of the ureteroscope.

It is worthy of note that the study does have some shortcomings. We used animal models instead of in vivo mannequins. Although animal models and human models have their own advantages, there are still slight anatomical differences between the use of the pig model and the human model like the ureters of pig may be more tortuous than those of humans, but the use of the pig model was effective in completing the preliminary study. Secondly, the pig model lacked the preliminary results of the lateral position surgery because it was difficult to immobilize the pig model during the lateral position surgery. This study only experimented on animals and conducted only stress tests, and the clinical situation is more complicated. For example, IRP estimations during actual FURS may be affected by the presence of renal stone fragments and turbidity in the renal pelvis. Therefore, future work is needed to further validate and improve the perfusion system through clinical applications.

## Conclusion

As a new idea for clinical practice, we propose a fiber-optic sensor-based system for testing IRP that can be done noninvasively and in real time. It is anticipated that urologists will benefit from this non-invasive and potentially cost-effective method for addressing high IRP in FURS. Although this system is currently a prototype for preclinical use, its potential impact on endourological devices and standardization of surgical skills holds promise for achieving favorable clinical outcomes.

## Materials and methods

### Ethical approval

The Animal Care Committee at Fujian Medical University approved the use of an anesthetized pig in accordance with all legal, public health, and ethical standards. (Issue No.: IACUC FJMU 2023-0178). All animal housing and experiments were conducted in strict accordance with the approved “Guide for the Care and Use of Laboratory Animals and also with arrive guidelines” of the institute.

### Self-designed patented devices

**Figure 4:**
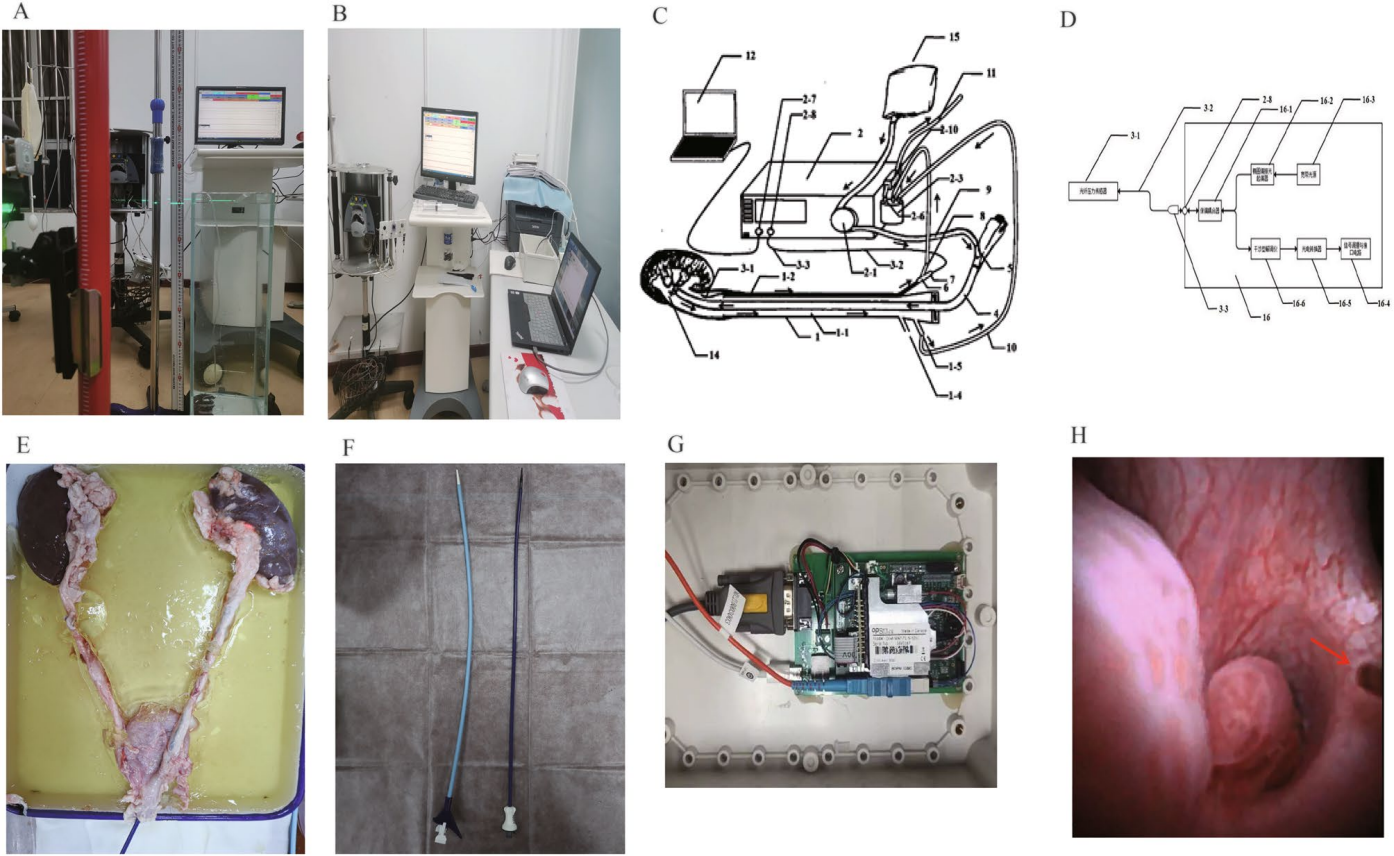
Experiments on precision and reaction time. [(**A**): Underwater pressure measurement at different depths. (**B**): Urodynamic instrument for pressure testing. (**C**): Conceptual diagram of a device for intrarenal pressure detection and control based on fiber optic pressure sensors. (**D**): Conceptual diagram of a pressure measurement device based on a fiber optic pressure sensor. (**E**): Isolated porcine kidney, ureter and bladder. (**F**): 12/14 Fr UAS (left) and 11/13 Fr UAS (right). (**G**): Fiber Optic Pressure Measurement System. (**H**): Fiber Optic Sensors for Pressure Measurement in the Kidney].

A numerical control system based on the monitoring of fiber optic pressure sensor on the side of the sheath for real-time control of the renal pelvis pressure during ureteroscopy, which consists of fiber optic pressure measurement system, independent dual-channel ureteral introducer sheath, numerical control platform with the function of perfusion/suction and intelligent terminal (Fig. 4). In the system, the flexible mirror is connected to the perfusion pump of the CNC platform through the main channel of the ureteral introducer sheath into the kidney, and the fiber optic pressure sensor enters renal pelvis through the side channel to monitor the renal pelvis pressure, and both channels can be connected to the negative pressure suction of the CNC platform. (CN109998698A/CN201910273371.X).

### Accuracy and sensitivity experiments

In the depth of 100 cm tank filled with pure water, the fiber optic pressure sensor and urodynamic pressure sensing device at the same time into the same water depth, and the pressure value of the measurement. Three measurements were taken at water depths of 10 cm, 20 cm, 30 cm, 40 cm, 50 cm, 60 cm and 100 cm.

Two isolated porcine kidneys were simultaneously placed into a fiber optic pressure transducer and a urodynamic pressure sensing device for intrarenal pressure and reaction time measurement. The intrarenal pressure was transiently altered by a short injection of large doses of water at a constant pressure of 10 cmH_2_0, 20 cmH_2_0, 30 cmH_2_0, 40 cmH_2_0, 50 cmH_2_0, 60 cmH_2_0, and 100 cmH_2_0 using a pressure fluid pump, and the response times of the two pressure sensing devices were obtained.

### Operative setup and technique

**Figure 5:**
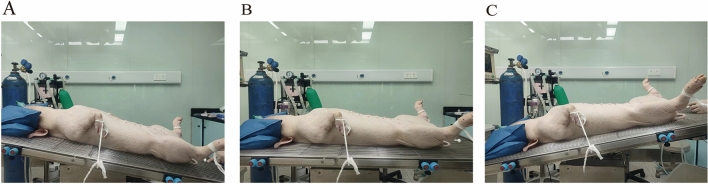
Surgical position [(**A**): Head-high-feet-low (15°), (**B**): Supine (0°), (**C**): Head-low-feet-high (10°)].

Six healthy, female pigs, 40–50 kg each, were sedated using Tiletamine Hydrochloride and Zolazepam Hydrochloride for Injection and intubated and then intra-airway anesthesia with isoflurane. Surgery was performed in the supine position and changes in body position were performed during the experiment (Supine: 0°, head-high-feet-low position: 15°, head-low-feet-high position: 10°, Fig. 5). The experiments were conducted according to the approved “Guide for the Care and Use of Laboratory Animals” of the institute.

A continuous and independent measurement was made in the UPJ and in the different calyxes (upper/middle/lower pole), in all pigs with different experimental conditions repeated in the same ureter of each pig. We used 11/13Fr and 12/14 Fr UASs which were placed at the UPJ and pelvis for this study. Through the UAS, a single-use digital flexible ureteroscope (Uscope 9.5 Fr, PUSEN, Zhuhai, China) was advanced to the renal pelvis. A 300 μm optical fiber for pressure measurement is then inserted. The frequency of fiber-optic measurements per second can be adjusted. The flow rate of 0.9% saline was set according to the flow velocity for continuous experiments (50 ml/min, 100 ml/min, 150 ml/min).

The perfusion pump provided a constant perfusion flow throughout the experiment, and the surgical operation was performed by the same group of surgeons. The fiber was positioned in the upper calyx, middle calyx, lower calyx or UPJ, testing was performed at intervals of 10 s in one minute.

### Statistical analysis

In this study, SPSS statistics v. 26.0 was used to perform the statistical calculations. Continuous variables are presented as mean ± standard deviation (SD). To assess the effects of manometry location, surgical position, and water flow rate, a one-way ANOVA was used. A paired t-test analysis was used to compare the accuracy, deviation, and response time of the 2 pressure measuring devices.

## Data Availability

The data that support the findings of this study are available from the corresponding author upon reasonable request.
